# Supplementary material for: Matrin3 (MATR3) Expression Is Associated with Hemophagocytosis
Source: Biomedicines. 2022 Sep 1;10(9):2161. doi: 10.3390/biomedicines10092161 (PMC9495864; doi:10.3390/biomedicines10092161)
Supplement: Supplementary file 1 [file biomedicines-10-02161-s001.zip › biomedicines-1872729-supplementary.pdf]

Supplementary Materials

Matrin3 (MATR3) Expression Is Associated with Hemophagocytosis

Wen-Chi Yang [1,2,*], Sheng-Fung Lin [1], Shih-Chi Wu [3,4] and Chih-Wen Shu [5]

[1] Division of Hematology and Medical Oncology, Department of Internal Medicine, E-DA Hospital, Kaohsiung 824, Taiwan
[2] Faculty of School of Medicine, College of Medicine, I-Shou University, Kaohsiung 824, Taiwan
[3] School of Medicine, China Medical University, Taichung 404, Taiwan
[4] Trauma and Emergency Center, China Medical University Hospital, Taichung 404, Taiwan
[5] Institute of BioPharmaceutical Sciences, National Sun Yat-sen University, Kaohsiung 804, Taiwan
* Correspondence: wenchi890079@gmail.com; Tel.: +886-7-6151101 (ext. 5005); Fax: +886-7-3906595

Supplementary methods:

1.1 Cell Culture

The THP1, acute myelomonocytic leukemia cell line, was cultured in RPMI medium (GIBCO, Life Technologies, Carlsbad, CA, USA) supplemented with 10% fetal bovine serum (FBS), 1% streptomycin and penicillin (P/S), 4.5 g/L glucose, 10 mM HEPES ((4-(2-hydroxyethyl)-1-piperazineethanesulfonic acid), 1 mM sodium pyruvate, and 1% β-mercaptoethanol. Puromycin (1 μg/mL) (Sigma, St. Louis, MO, USA) was added for stress selection of THP1 cells infected with shRNA empty vector and shRNA-MATR3 lentivirus. Approximately 1×10^5 cells were plated in 10 cm dishes and cultured at 37 °C in a humidified atmosphere containing 5% CO_2. The native cell line was purchased from the Food Industry Research and Development Institute, Taiwan, in 2012, May. The THP1 cell line was confirmed to be mycoplasma-free but was not authenticated. However, we amplified cells when we received and used the initial stocked cells to do the study.

1.2. mRNA Isolation and Expression

Bone marrow (BM) was obtained by sternal or posterior iliac bone aspiration and collected into heparin tubes containing 60 USP units of sodium heparin. Total RNA was extracted from BM samples of the enrolled patients and MATR3-KD THP1 cells using Trizol (Invitrogen, Life Technologies). The RNA input (2 μg) for cDNA synthesis was determined by measuring OD_{260}. RNA was transcribed to first-strand cDNA by using TaqMan® High Capacity Reverse Transcription Kit (Applied Biosystems, Life Technologies), according to the manufacturer's instructions.
The cDNA sequences of *MATR3* and *NF-κB* were evaluated, and the specific forward and reverse primers and TaqMan® probe were designed using Primer Express software version 1.5 (Applied Biosystems, Life Technologies). The TaqMan® MGB probe was synthesized and labeled with fluorescein amidite (FAM) fluorescent dye (Applied Biosystems, Life Technologies).
mRNA expression levels of *MATR3* and *NF-κB* (two sets of each gene) were analyzed by qRT-PCR using the following primer sets and probes.
MATR3-1:
Forward primer 5´-GACACATGCAGAAAGGCAGA-3´,
Reverse primer 5´-ACGCTGGTGGTGTGGTTGTGTAA-3´,
TaqMan® MGB probe 5´[6FAM]AACCACAGAGGATGCTCAGG[TAM],
MATR3-2:
Forward primer 5´-TGGAGCAAGTCACAGTCGTC-3´,
Reverse primer 5´-TCCTTGCAGGTTTCCATTTC-3´,

TaqMan® MGB probe 5´ [6FAM]TGGACCAAGAGGAAATCTGG[TAM].

NF-κB-1:

Forward primer 5´-CTGGAAGCACGAATGACAGA-3´,

Reverse primer 5´-TGAGGTCCATCTCCTTGGTC-3´,

TaqMan® MGB probe. 5´ [6FAM]GCCTATTTGCAAGCAGAAGG[TAM]

NF-κB-2:

Forward primer 5´-ACAAATGGGCTACACCGAAG-3´,

Reverse primer 5´-ATGGGGCATTTTGTTGAGAG-3´,

TaqMan® MGB probe. 5´ [6FAM]CAGTGGTGCCTCACTGCTAA[TAM]

All the reactions were carried out in a final volume of 25µL containing 200 ng of cDNA (as total input RNA), 400 nM of each primer, 200 nM of the probe, and 12.5µL of 2X TaqMan®Universal PCR Master Mix (Applied Biosystems, Life Technologies). qRT-PCR was performed in an ABI Villi 7 Sequence Detector (Applied Biosystems, Life Technologies), employing the following PCR cycling parameters: 95 °C for 10 min, followed by 40 cycles at 95 °C for 20 s and 60 °C for 1 min. The expression levels of the *MATR3* and *NF-κB* genes were normalized to the internal control β-actin to obtain the relative threshold cycle (ΔC_T). The C_T values of β-actin were controlled between 18 and 22.

Supplementary Tables:

Table S1. Correlation of genes expressions in PBMCs and BM cells[&.]

[&] only 26 patients who had both PB and BM samples<br>**Pearson correlation**	**PBNFκB-1**	**PBNFκB-2**	**PBMATR3-1**	**PBMATR3-2**	***P-value***
**BMNFκB-1**	0.231				*0.302*
**BMNFκB-2**		-0.117			*0.429*
**BMMATR3-1**			0.068		*0.777*
**BMMATR3-2**				0.098	*0.658*

Table S2. Correlation of gene expressions in PBMCs

**Pearson correlation**	**PBNFκB-1**	***P-value***	**PBNFκB-2**	***P-value***	**PBMATR3-1**	***P-value***	**PBMATR3-2**	***P-value***
**PBNFκB-1**	1		-0.017	*0.91*	0.061	*0.683*	0.043	*0.776*
**PBNFκB-2**	-0.017	*0.91*	1		0.280	*0.044**	0.31	*0.025**
**PBMATR3-1**	0.061	*0.683*	0.280	*0.044**	1		0.768	*<0.001**
**PBMATR3-2**	0.043	*0.776*	0.31	*0.025**	0.768	*<0.001**	1	

**P* < 0.05, statistically significant*

Table S3. Correlation of gene expression in BM cells

**Pearson correlation**	**BMNFκB-1**	***P-value***	**BMNFκB-2**	***P-value***	**BMMATR3-1**	***P-value***	**BMMATR3-2**	***P-value***
**BMNFκB-1**	1		0.587	*0.003*	0.240	*0.281*	0.423	*0.044**
**BMNFκB-2**	0.587	*0.003**	1		0.702	*<0.001**	0.783	*<0.001**
**BMMATR3-1**	0.240	*0.271*	0.702	*<0.001**	1		0.741	*<0.001**
**BMMATR3-2**	0.423	*0.044**	0.783	*<0.001**	0.741	*<0.001**		

**P* < 0.05, statistically significant*

Supplementary Figures

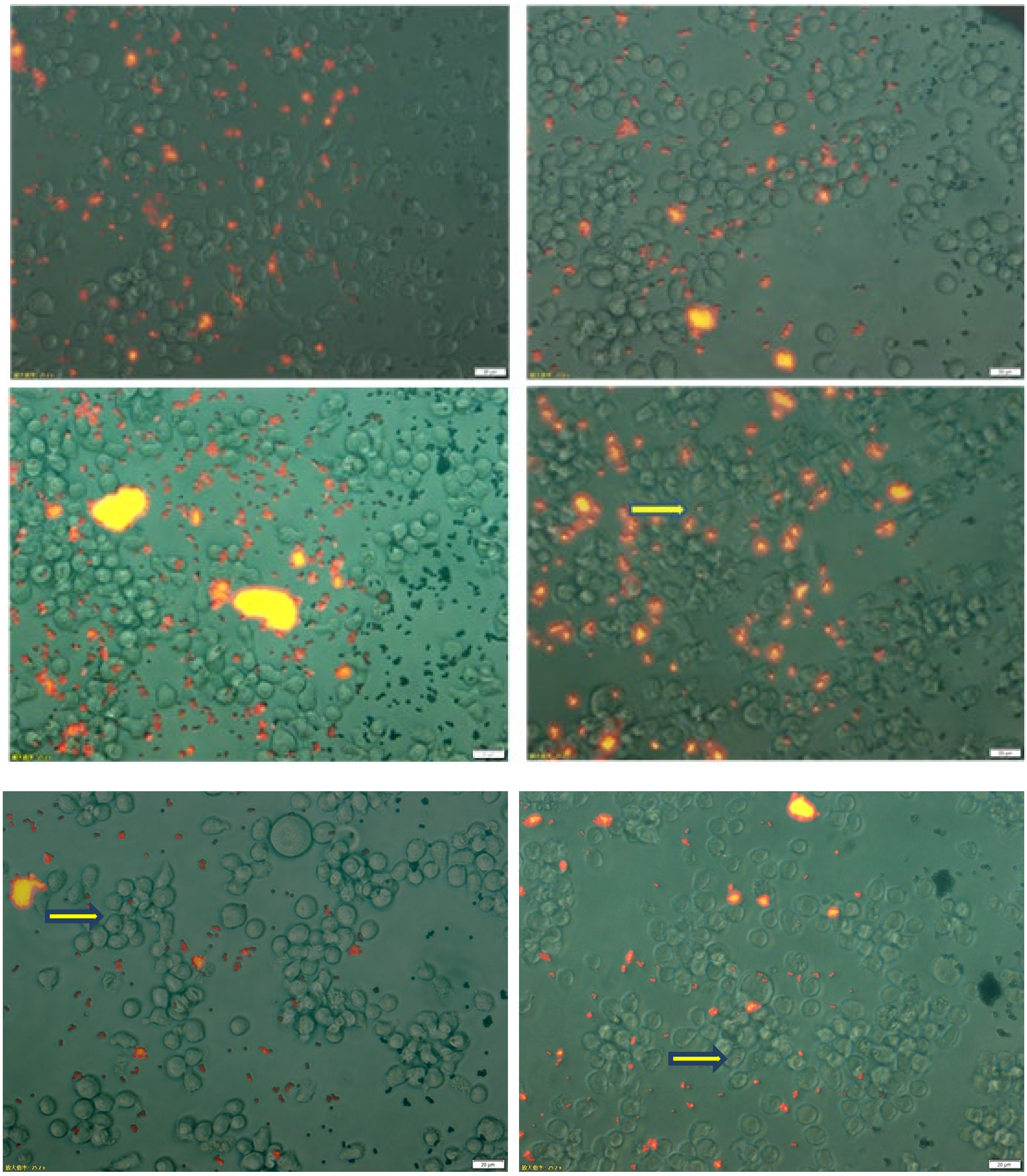

Figure S1. Red fluorescent 0.5 μL carboxylate modified polystyrene latex beads co-culture with *shRNA-MATR3* KD THP1 cells for 30 minutes. Upper: shRNA control vector transfected THP1 cells. Middle: *shRNA2-MATR3* KD THP1 cells. Lower: *shRNA4-MATR3* KD THP1 cells. Yellow arrow: Phagocytosis of latex beads by THP1 cells.

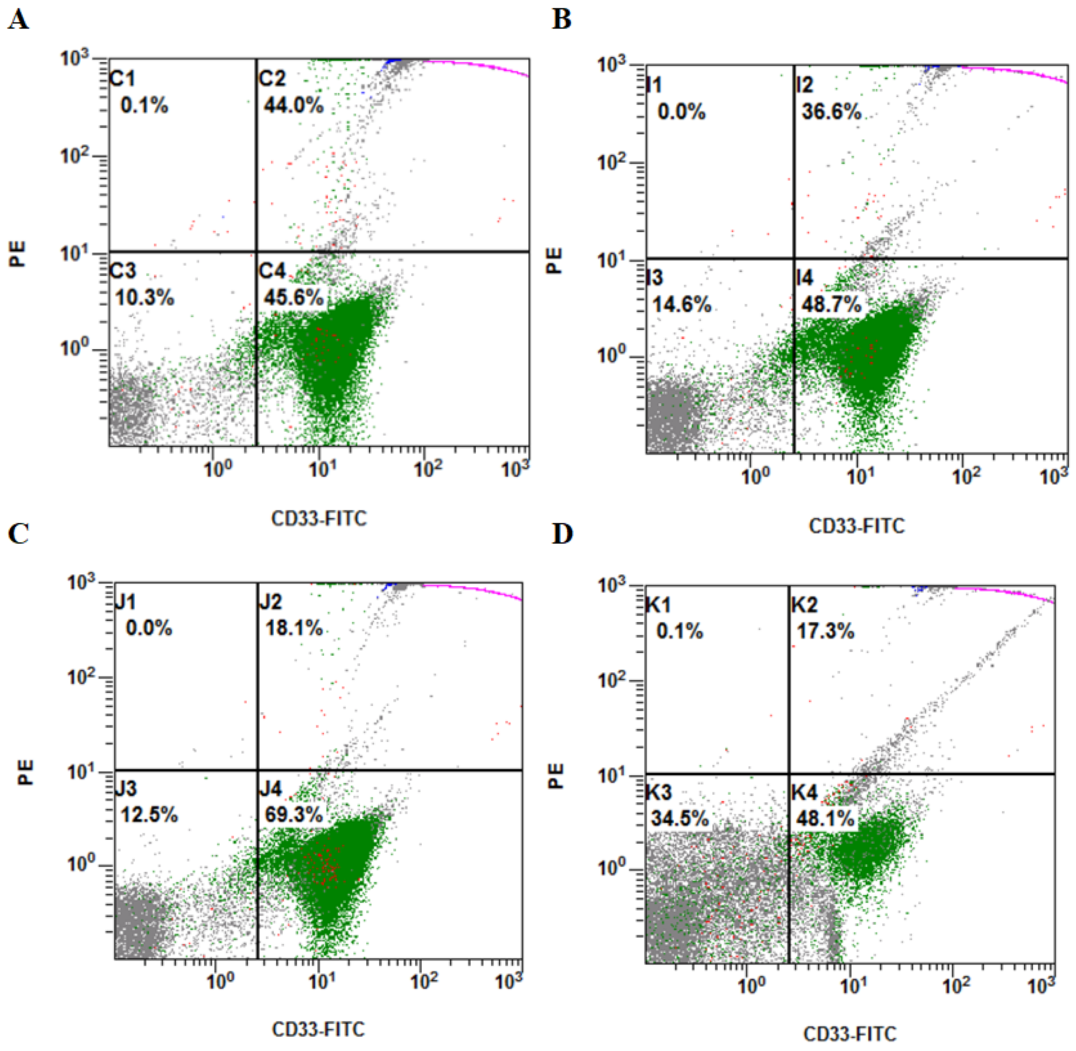

Figure S2. Red fluorescent 0.5 μL carboxylate modified polystyrene latex beads co-cultured with *shRNA-MATR3* KD THP1 cells for 3 h. (a) *shRNA2-MATR3* KD THP1 cells (b) *shRNA4-MATR3* KD THP1 cells. (c) shRNA control empty vector transfected THP1 cells. (d) naïve THP1 cells. Live THP1 cells are CD33-FITC positive.

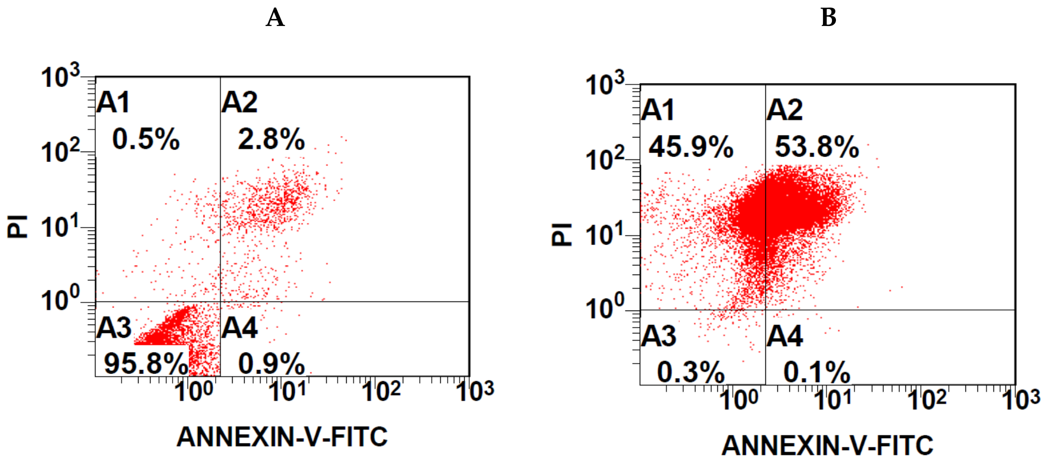

Figure S3. Annexin V expression (apoptosis) of Jurkat E6 cells (**A**) before and (**B**) after culture at 56 ℃ for 30 min.
